# Evidence-based research impact praxis: Integrating scholarship and practice to ensure research benefits society

**DOI:** 10.12688/openreseurope.14205.1

**Published:** 2021-11-16

**Authors:** Eric A. Jensen, Mark Reed, Aaron M. Jensen, Alexander Gerber

**Affiliations:** 1 Institute for Methods Innovation, 1752 Charles Avenue, Arcata, CA, 95521, USA; 2 University of Warwick, Gibbet Hill Road, Coventry, CV4 7AL, UK; 3 Qualia Analytics, Unit 23, Trinity Technology & Enterprise Campus, Pearse Street, Dublin, D02 WR66, Ireland; 4 SRUC Aberdeen, Ferguson Building, Craibstone Estate, Bucksburn, Scotland, AB21 9YA, UK; 5 Institute for Science and Innovation Communication, Briener Str 25, Kleve, 47533, Germany; 6 Rhine-Waal University, Marie Curie Str 1, Kleve, 47533, Germany

**Keywords:** Research impact, evidence-based research impact, impact development

## Abstract

Effective research impact development is essential to address global challenges. This commentary highlights key issues facing research impact development as a nascent professional field of practice. We argue that those working on research impact should take a strategic, ‘evidence-based’ approach to maximize potential research benefits and minimize potential harms. We identify key features of evidence-based good practice in the context of research impact work. This includes integrating relevant research and theory into professional decision-making, drawing on a diversity of academic disciplines offering pertinent insights. Such an integration of scholarship and practice will improve the capacity of research impact work to make a positive difference for society. Moving the focus of research impact work to earlier stages in the research and innovation process through stakeholder engagement and anticipatory research can also boost its effectiveness. The research impact evidence base should be combined with the right kind of professional capacities and practical experience to enhance positive impact. Such capacities need to be developed through relevant education and training, for example, in participatory methods and social inclusion. Such training for research impact work needs to forge strong links between research impact scholarship and practice. Finally, there is a need for improvements in the evidence base for research impact to make it more practically useful.

## Plain language summary

Researchers are often deeply committed to making a difference in the world. Achieving beneficial outcomes for society involves professional work aimed at creating such positive impacts from research (i.e., ‘research impact’). A field of professionals dedicated to developing research impact has been emerging in several countries globally. This essay argues that these professionals can work with researchers in new and better ways to extend the impact of research. For example, they can assess whether public needs are really being addressed by planned research and innovation initiatives. To maximise the value of research impact work, existing knowledge should be more effectively mobilised. Those devoted to making a difference using research should work closely together, integrating academic and practical expertise and experience. Working together and applying the best available evidence to this kind of work will benefit all involved, most importantly the public.

## Introduction

Leveraging the knowledge to make the world a better place is a noble goal in research. However, it can also be challenging to develop the most appropriate strategies for non-academic impact objectives, intended beneficiaries and specific economic, social and cultural conditions. Faced with numerous challenges, we argue in this commentary that the emerging field of professional practice around the generation of research impact would benefit from more evidence-based approaches, where appropriate scholarship and professional practice are brought together into a coherent praxis. An evolution towards evidence-based research impact praxis is essential for progress in how research impact professionals operate. This essay sets out how research impact professionals and scholars can work together to develop improved strategies and practices. We argue that better, more socially responsible development and application of the best available scholarship will deliver more beneficial processes and outcomes both for society and for the research enterprise.

Research impact as a recognizable field of professional practice is relatively new, overlapping with other more longstanding research-related practice domains such as science communication and sustainability. This newer field has been developing differently around the world. Job titles such as ‘impact officer’ have become commonplace in some countries, such as the UK, in the last decade. In this essay, we use the term ‘research impact professionals’ to refer to those involved in managing or developing research impact, both as their primary employment (for example, impact officers) and as researchers who are also working to generate impact beyond the academy as a secondary aspect of their work. Non-academic impact aims to extend beyond academics and students to include industry, policy makers and different types of publics such as young people, migrants, or orthodox religious groups.

This is an aspect of research and innovation systems that has recently come to the fore, riding the wave of top-down initiatives to promote non-academic impact, initially as a condition of research funding and, more recently, integrating into national research assessments. Example initiatives in different countries include the UK’s Research Excellence Framework (REF), Italy’s Research Quality Evaluation, the Spanish National Commission on the Evaluation of Research Performance, Hong Kong’s Research Assessment Exercise and Australia’s Engagement and Impact Assessment ( Reed *et al*., 2021).

However, there is growing evidence that top-down initiatives to promote impact beyond the academy may introduce unintended negative outcomes within the research and innovation systems. When coupled with limited research impact capacity or expertise within the institutions charged with delivering benefits to society, prominent negative outcomes may emerge. Examples of such unintended outcomes may include forms of de facto corruption such as elite capture ( de Hoop *et al*., 2016) and conflicts of interest ( Chubb & Reed, 2018; Watermeyer, 2019). The European concept of “RRI” (Responsible Research and Innovation) has similarly been criticized for being more of a policy prescription than a paradigm that is also supported widely and implemented from the bottom up ( Gerber *et al*., 2020). Additionally, forms of cultural imperialism may extend the prevalence of social inequities and opportunity costs for the wider populations who engage but receive little or no benefit ( Cooke & Kothari, 2001; de Vente *et al*., 2016; Watermeyer, 2019) from the research and innovation system. 

To ensure research impact scholarship provides more relevant insights for the community of practice in this domain, there is an ongoing need to engage with intended beneficiaries and identify the impact goals or outcomes that will drive the evaluation ( Jensen, 2015b). Reed *et al*. (2021:3), in their characterization of research impact as “demonstrable and/or perceptible benefits…”, emphasize the subjectivity of benefits that may arise from research, particularly when the benefits or advantages to one group may be perceived as damaging or harmful to the interests of another group (or the same group in a different time or context). Furthermore, situations may arise where the interests of beneficiary groups are poorly represented, and over-managed participatory processes may lead to forced agreements that simply maintain existing power structures ( Cooke & Kothari, 2001).

The challenge here is partly structural, based on funding and incentive structures and the types of research that tend to be prioritized. Indeed, few research funding schemes incentivize genuinely co-productive research with diverse stakeholders. Limited co-production between researchers and practitioners can be a major barrier to impact, leading to the generation of unintended consequences for interests of groups who were not engaged or were not able to engage effectively in the process ( Adams, 2008; Cooke & Kothari, 2001). For example, Fritsch & Newig (2012) conducted a case-survey meta-analysis of environmental management publications involving stakeholder participation (many of which were initiated and written up by researchers) and found a bias in outcomes towards the interests of over-represented groups, typically at the expense of already-marginalized groups. Such outcomes can damage the trust between research institutions and marginalized groups, with long-term negative consequences for both sides. Instead, researchers and practitioners need to consider the sometimes-competing interests of different groups as they change over time in response to changing social and political contexts or changes in the personal circumstances of participants ( Sarkki *et al*., 2014), to avoid over-representing those most easily accessible to researchers ( Colvin *et al*., 2016) and represent the diversity of perspectives and realities voiced by different groups ( Moon *et al*., 2019).

Existing scholarship on participatory processes argues for the importance of giving power back to intended beneficiaries in an attempt to reshape the desired outcomes and terms of an evaluation based on their perceptions (e.g., Estrella & Gaventa, 1998; Guijt *et al*., 1998; Villaseñor *et al*., 2020). Drawing on research and evaluation methods from the social sciences and the arts and humanities, the participatory process emphasizes the value of inclusion by ensuring a plurality of voices and perspectives. Specifically, the process of making research relevant for intended beneficiaries should give marginalized voices weight when deciding what beneficial outcomes count as research impact ( Coemans *et al*., 2015; Reed *et al*., 2021; van der Vaart *et al*., 2018).

As the policy drive for ‘impact’ spreads and gains strength ( Edler *et al*., 2012; Oancea, 2019), the cadre of research impact professionals employed to support the development of non-academic impact continues to expand, alongside the quantity of researcher time and resources dedicated to impact work as a secondary or tertiary activity ( Jensen, 2020b; Jensen & Holliman, 2016; Wróblewska, 2021). However, this expansion of research impact staff is not yet accompanied by formal training, such as the development of master’s degree programmes or widely recognized certification schemes that could underpin a shared understanding of professional practice in this domain. This paucity of formal training raises concerns about where these research impact officers are supposed to look for insight and guidance about effective professional practice.

A loose collection of academics, consultants and representatives from government and non-governmental agencies, funding organisations and private companies offer research impact advice while drawing on a diverse range of backgrounds and expertise. The need remains to clarify and develop a coherent framework, shared understanding and working consensus among these actors of professional practices to most effectively identify, evaluate and evidence impact within the research and innovation system, while minimising the potential for unintended outcomes and risks of harm and improving societal benefits.

Here, we argue that this need for professionalization in research impact work is best addressed through an integrated approach with support in scholarship and practical research experience.

## Key challenges

With the growing expectations for research impact work, both professionals and scholars in this domain must further develop their capabilities for critical self-reflection, evidence-based practice, and robust impact evaluation. Indeed, the development of these capabilities will underpin necessary long-term progress for research impact policy, practice and scholarship. Across our diverse fields, we have been involved in research impact practice and scholarship as these domains evolved over the years. In our work at the interfaces between policy, practice and scholarship, we have helped address numerous and varied challenges that we, and many other scholars and practitioners, encounter relating to research impact processes. We have previously highlighted a range of these challenges (for example, Chubb & Reed, 2018; Jensen *et al*., in press; Reed *et al*., 2018; Reed *et al*., 2021; Vella *et al*., 2021), but it is clear that as a starting point scholarship in this domain needs to be more relevant to practice.

### Making impact scholarship relevant

First, there is the challenge of making research impact scholarship relevant to professional practice and intended beneficiaries. Few academic publications on the theme of research impact attempt to establish why this work matters for professional practice, nor explain how to address results and findings. Moreover, findings, insights or implications presented in academic publications may remain inaccessible to practitioners when obscured by disciplinary academic jargon and opaque writing.

Faced with such communication challenges, participatory and evaluation processes can be practical and valuable ( Boydell *et al*., 2012) when clarifying the relevance of research topics that are sensitive or hard-to-verbalize. These processes can also support dialogue with those the research intends to benefit, thereby facilitating more fruitful knowledge exchange. However, maintaining such dialogues efficiently, for example by using evaluation surveys, can be challenging. Evaluations require that feedback is listened to, acted on, and changes are made to address inadequacies. Participatory approaches often work with small numbers of people, but it may be essential to broaden the number of informants to clarify the relative prevalence of key issues that impact the welfare of a larger population. Extending the evaluation of success indicators from small to large samples requires complementary tools and resources for more quantitative measures of change over time (e.g., see Morgan, 2007; Morgan, 2014). Inspired by Heneghan *et al*. (2017), we emphasize the need for research impact scholarship to provide relevant, accurate and timely insights that practitioners can implement.

### Making impact scholarship accessible and applicable

Once the relevance of research impact scholarship is clarified, it is necessary to ensure findings and insights are accessible to practitioners. Although open data and open methods are more common with recent advances across research and innovation systems (e.g., Piwowar *et al*., 2018), many research findings are primarily published in English and behind paywalls. Furthermore, efforts to translate findings from impact scholarship into non-English languages are currently limited and ad hoc, leaving significant scholarly contributions inaccessible to many global researchers and practitioners in non-English speaking and low-income countries. A prerequisite for this would be the applicability of theoretical frameworks and assessment methods in the communities of practice. Applicable or applied research can lead to direct actions or solutions that address the specific needs of intended research beneficiaries in a range of contexts. In contrast, basic research may help to define or describe problems but may seem more abstract or conceptual, appear less relevant to practice and be more challenging to demonstrate how it can be applied. Indeed, the majority of published research describes or defines problems (known as “mode 1”), often proposing and testing hypotheses aimed at leading to a generalisable theory that is broadly applicable across many different contexts. The main alternative is research that focuses on the more localised experiences of individuals or groups in specific contexts or situations (known as “mode 2”) in which knowledge is generated. Recommendations based on narrow individual case studies may overgeneralize beyond the context in which the study was originally conducted. Scholarship should work on clarifying the conditions for transferability of research findings, rather than assuming universal applicability. Collaboration between scholars and practitioners will help to improve the relevance and applicability of research impact findings.

Considering how much both the rationale and the assessment of non-academic impact are increasingly interwoven with the respective research systems and cultures, research funding processes greatly affect research impact work. Formal review procedures organized by funding bodies and even regulatory initiatives by science policymakers impinge on definitions of impact, as well as the perceived legitimacy of different research impact goals and approaches. Indeed, reviews of grant applications and funding decisions comprise key moments where policy meets practice, but the role of research impact scholarship in such concrete tasks is limited or non-existent in our experience. Academic methods and models used in research impact scholarship need to be made applicable in ways that allow a direct transfer into these kinds of specific, critically important tasks. Further changes to who reviews proposals and projects may be needed as those reviewers “need to be selected, briefed and possibly even trained with regard to their capability to assess different degrees of engagement and participation” ( Gerber, 2018, p. 2). Practically speaking, the key applicable points from scholarship relevant to non-academic impact will need to be operationalized in the form of criteria for proposal evaluation, reviewer selection and reviewer briefings, guidance for grant-writers and systemic changes and infrastructure needed to make research impact more effective and inclusive.

### Ensuring and increasing the quality of impact scholarship

Research impact scholarship needs to avoid questionable practices that could produce errors and undermine accuracy ( John *et al*., 2012) in findings and implications. There is a risk that research impact scholarship can have errors, mistakes, or inaccuracies that are subsequently applied in practice, leading to unintended or undesirable outcomes. There are, for instance, calls to extend research methods from other disciplines, such as medicine, that include standardizing sets of core measures or indicators that can be consistently reported and enable meta-analysis ( Nichols *et al*., 2021; Stewart *et al*., in prep.). Despite some notable initiatives in specific impact-relevant domains (consider the work of Conservation Evidence in environmental science; Sutherland *et al*., 2015), there are very few systematic reviews of research impact scholarship.

Evidence synthesis, systematic reviews and meta-analysis that compare research designs, methods, results, and findings across studies can provide a more reliable basis for recommending changes in practice. Steps to increase research quality also help ensure that methods and results become more comparable across studies and are generalized correctly. This will improve the probability that efforts to make such scholarship more relevant and applicable will not be in vain. In contrast, a lack of comparability hampers generalizability and thus again the applicability of the scholarship at large. Many evaluations of research impact lack key methodological details and do not even meet the quality criteria for being included in systematic reviews. Most commonly, researchers trained in the natural or physical sciences attempt to use social science methods to evaluate impact, including surveys, interviews and focus groups. However, there are often shortcomings in the research design, methods, and analysis that limit rigour from these studies. For example, quantitative evaluations may lack adequate sample sizes for statistical power, replication, baselines, or control comparison. Whereas qualitative evaluations may lack sufficient triangulation between sources or critical interpretation of findings (e.g., Jensen & Laurie, 2016; Jensen, 2020b).

Karcher *et al*. (under review) have recently completed one of only a few systematic reviews of impact evaluations. Findings from this review have shown that evaluations often referred to the evidence of research impact as outcomes that create *products* (e.g., reports, maps, tools), enhance the *usability of knowledge* (e.g., credibility, salience, legitimacy) or improve *social connection* (e.g., networking, awareness, learning, trust-building) between stakeholders. While the objectives of evaluated interventions often aimed to achieve policy, economic and societal impacts, evaluations rarely collected evidence on these outcomes. These results may represent a failure of research to generate impact or reflect shortcomings in impact evaluations in the available published literature, including methodological limitations (see Jensen, 2020c) and misalignment between the evaluation timescales over which impacts occur ( Gow & Redwood, 2020; Morris *et al*., 2011).

## Evidence-based pathways toward research impact

In our view, there are several concrete measures that science policy and research funders, universities and even stakeholders can take to foster a more evidence-based and thus effective research impact:

- The praxis of applying scholarship on how to anticipate and influence non-academic impact of research and innovation systematically, will foster more effective and resource-efficient impact actions. Growing policy expectations for research impact will most likely drive further professionalization to design and implement more evidence-based approaches. This will make both the impact activities and their evaluation methodologically more robust.
- For practitioners to explore and potentially apply the existing body of evidence, the relevant scholarship needs to be conveniently accessible. Since the scholarly publishing system is unlikely to make the required changes to deliver this voluntarily, it will be imperative that research funding organisations further incentivize not only open access but also open data and open methodology. This would foster the comparability and generalizability of available evidence and encourage systematic reviews that sift through the body of research and provide professionals with more quality-assured evidence they can use.
- It is also a prerequisite for this praxis to be addressed from both sides: by impact professionals being reflective and open to potential changes in established practices, and by impact scholars ensuring direct applicability of their findings. The reflexivity in practice must include honest self-assessments of the limitations of one’s work so that evidence that potentially invalidates previous practices will not be rejected outright. Ideally such praxis would evolve from mutual learning through collaborative action research, and by sharing experiences that may benefit the wider community of practice.
- All pathways toward more evidence-based research impact will also need to increase awareness among funders, research performing organisations and individual researchers of the need to anticipate and act upon the potential benefits and harms of the research and innovation they are pursuing. Taking well-designed steps to involve stakeholder perspectives early in the research and innovation process can pay dividends in long-term impact. This is where research stakeholders in general, and the potential end users of technological and social innovations in particular, must be empowered to voice their needs and expectations in a way that can have a real influence.
- In response to this increased awareness, impact professionals will require significantly more capacity building than is even offered now. Whether integrated into the syllabi of existing professional development, master’s or PhD programmes, or offered as stand-alone programmes, it should go without saying that more evidence-based approaches can only be fostered by similarly evidence-based teaching and training, which is not the case yet.
- One of the key aspects of such training must be to highlight the importance of actively including marginalized groups in the design and implementation of research impact actions using evidence-based communication and involvement strategies (see Jensen, 2013; Kennedy *et al*., 2018). Existing scholarship on social inclusion from academic fields such as sociology offers a wealth of untapped insight that can make practice more effective.
- In addition to professionalizing research impact work as such, the activities in this field will also need to be monitored and evaluated more systematically, and ideally also more comparatively ( Jensen, 2014; Jensen, 2015a). This assessment needs to be of sufficient methodological rigour (e.g., Jensen & Laurie, 2016; Kennedy *et al.*, 2021), and ensure that appropriate ethical principles are considered, such as informed consent for participation and responsible data protection and management ( Jensen, 2020a). Maintaining transparency and openness regarding the nature of funding and its organizations and institutions, can have a positive influence on the design of research impact activities (see Gerber, 2014).
- Ensure resource-efficiency to maximize opportunities for positive research impact activities.

We recognize that the suggested pathways forward will always be affected by the perspectives of researchers, practitioners and intended beneficiaries of research impact activities. These perspectives will also be influenced by institutional, local, and cultural circumstances.

## Conclusions

In this commentary, we have expressed concern that research impact professionals may not sufficiently benefit from *relevant*, *accessible*, *applicable,* and *quality* scholarship. We contend that this is an ideal time to consider the trajectories of research impact before problematic professional norms in this still-forming field become too ingrained. However, the domain of research impact is not yet an established ‘field’ in the conventional sense. Indeed, it is still a loosely developed community of practice that comprises researchers, various research support officers and other professionals and staff at research funding organizations. While this status for the domain of research impact may make our call for evidence-based practice seem premature, we believe this is a meaningful discussion that must involve research impact stakeholders and those who work across research-practice boundaries.

This commentary aims to nurture reflectiveness in this community of practice by starting a conversation about the effectiveness of impact-related practice, evaluation and scholarship. In our view, much ‘evidence’ in the domain of research impact will need to be challenged and considered provisional for quite some time. This provisional nature of findings will often happen at the frontline of evolving areas of scholarship. In the meantime, there are many well-established and well-evidenced insights and theories that can be safely used to underpin evidence-based research impact work.

While RFOs today may be satisfied with a general increase of ‘any’ non-academic impact, regardless of why and how those materialize, their expectations are certainly growing. At the same time, other societal stakeholders are increasingly demanding their voice be heard further upstream in the research and innovation process. In response to the combination of these top-down and bottom-up trends, capacity building initiatives are slowly starting to respond. Research and impact professionals will increasingly be trained in assuming a more anticipatory approach to analyse and account for the risks and benefits of their research and innovation. This can empower them to plan and implement more appropriate impacts, for instance avoiding detrimental implications and bolstering positive outcomes for marginalized groups.

More than just ascertaining and appreciating non-academic impacts, RFOs can (and must) increasingly demand a more formative and thus evidence-based approach. Impact officers could, for instance, learn to professionally manage focus groups with stakeholders already during the research design phase. For the time being, most policy frameworks, such as the Research Excellence Framework in the UK insufficiently incentivize such upstream models by requiring that impact evidence be linked to specific research outputs (most often, journal articles or books). Such incentive structures have to be very carefully calibrated to ensure that they are rewarding long-term pro-social outcomes.

We argue that collaboration between impact scholarship and practice will improve the relevance and transferability of research impact. Furthermore, improved quality in the available evidence will offer the most significant practical foundation for improving research impact work, now and in the future. We are inspired by Heneghan *et al*. (2017) when we emphasize that the direction for research impact scholarship should seek to provide relevant, accurate and timely insights that practitioners can implement.

We intend to ignite further discussions about the principles and practices of evidence-based research impact. We also want to address the everyday challenges and experiences of using current evidence to inform and expand research impact development. This is the only way that evidence-based research impact can live up to its potential in a world where it is increasingly needed.

## Key messages

1. Professionals working in the emerging field of ‘Research Impact’ can make their activities more effective by applying relevant evidence from the social sciences to improve their strategies and methods. As prerequisites, relevant scholarship on research impact must be identified and made accessible. Scholars in this domain should extend efforts to make their work directly applicable to practice. Professionals will need to be reflective and open enough to consider changing established practices if necessary. Ideally, scholars and practitioners could collaborate to enable an evidence-based research impact praxis.
2. Stakeholder groups must be empowered to voice their needs and expectations, for instance, potential end-users of technological innovations. It is crucial that these changes are set in motion before problematic professional norms in this still-developing field of practice become solidified. Research funding organisations (RFOs) play a key role in incentivizing responsible approaches both to open science and research impact.
3. Capacity building for more evidence-based research impact should be integrated into research training at postgraduate level, and perhaps even in undergraduate programmes. Both policy and practice need to develop the capability to ensure that marginalized groups are actively involved at an early stage of the research impact process. It will also be imperative for RFOs to train and guide research applicants, reviewers and evaluators in how to meet their impact expectations. In general, all impact activities need to be evaluated more systematically with sufficient methodological rigour and consideration of ethical principles.

## Data availability

All data underlying the results are available as part of the article and no additional source data are required.
