# Supplementary material for: Evidence-based research impact praxis: Integrating scholarship and practice to ensure research benefits society
Source: Open Res Eur. 2023 Aug 4;1:137. Originally published 2021 Nov 16. [Version 2] doi: 10.12688/openreseurope.14205.2 (PMC10884597; doi:10.12688/openreseurope.14205.2)
Supplement: Peer review history

# Reviewer report: Reviewer response for version 2

**DOI:** 10.21956/openreseurope.16766.r37078

**Published:** 2024-03-11

**Authors:** Kathryn Watson

**Affiliations:** 1 Business School, Leeds University, Leeds, England, UK

This article addresses the gap between academia and practice and the professional development of individuals who span both domains. I felt a little unclear as to who is the intended audience for this paper. Is it about, and targeted at, professional staff working in academia in roles similar to myself, or practitioners that have become involved in academia, perhaps as Professors of Practice or of Research Impact, or external practitioners in organisations and institutions that engage with the outputs of academic research?  

 Overall, I think there are some edits that could be made to this paper to give it more clarity for reader. Having said that it did make me think about professionalization of the roles of people working in support of research impact and the training that would benefit them.  Also, how do you recruit, induct and develop people in the type of adjunct roles that span both academia and practice or policy? I would agree that there should be greater recognition of impact support roles which will benefit both academia and their external research impact beneficiaries.

**Clarity around terms and focus:** The terminology used in discussions related to research impact has been raised by the first reviewers and the article now comments on the inconsistent terminology used in the domain. However, the paper could be more explicit and precise and this would greatly help the reader. For instance in the Plain language summary the phrase ‘Those devoted to making a difference using research’ is vague and I think that, overall, this summary could give a much clearer picture of what the paper is about. While some amendments have been made following the first two reviews, I think there is more that could be done that would give the paper greater impact with its intended audiences.

 I would recommend inserting a small table with some definitions of terms.  I have a query about the term ‘evidence-based research impact praxis’ (referred to in the title and on page 3) and what it is that the authors are advocating here – are you trying to make impact from research more relevant or are you trying to fundamentally change how academics do research? In the Conclusions you refer to ‘evidence-based practice’ which is obviously not the same.

 In the subsequent paragraph on page 3 – with the existing definitions, all research academics could be described as ‘research impact professionals’ but I assume that is not the intention. 

**Framework and principles:** David comments that there is never likely to be a coherent framework widely accepted and practiced but there could be some principles that will translate across many contexts and recommends to think of principles rather than a framework.  I think this is a very useful suggestion. As both reviewers have commented, there are considerable differences across disciplines both in what is academic scholarship and rigor and what is impact. So, some generic cross-disciplinary principles would be useful and have the benefit of broader applicability. At the moment I find that the paper is focused on the expertise of the authors (evaluation, participatory research) and while I appreciate that they want to promote these approaches, they could recognise that there are other perspectives/approaches that maybe also be valid. If the authors are seeking to provide some principles that can benefit academia widely then I think they should look more deeply at what works (and does not work) in different areas.

 I would add to this that the nature of impact across disciplines is vastly different and in this context I find it hard to believe that there is a generic approach to professional development around research impact that can be applied and recognised across all disciplines.  Showing how your methods could work in other areas would have value, (see comment regarding examples below).

**Specifics and real-world examples:** I agree with David that the commentary lacks real world examples of linkages between scholarship and practice. In advocating participatory research approaches, the paper could give some examples of how this approach has been used successfully in different disciplines, perhaps including STEM areas. Also, the point has been made that much impact comes out of discipline specific research and I think that there is not enough recognition in the paper that other approaches are valid and can have considerable impact, without necessarily being participatory or involving co-production. For instance, much funded research does require a significant element of collaboration and partnership with the intention of generating non-academic impact – this does not necessarily mean that the collaborators are actively involved in the implementation of the research. Currently I don’t think that the paper gives sufficient recognition and tangible examples of how impact scholarship varies across specific disciplines and how practitioners can access and engage with those different contexts. 

 Practitioners also often seek expertise from interdisciplinary teams and while there might be the desire within institutions to work in this way there are still many barriers to effective interdisciplinary research that can solve real world problems. How can the development of impact professionals help address these challenges?

**Training and professionalism:** Elyse comments that different forms of expertise are needed at different stages in the process. The authors might clarify *where in the system* they believe the training and professionalization efforts are likely to have the greatest impact –e.g. more faculty with translation skills, expanding professors of practice, deepening commitment to professional staff to work with faculty. David points out that impact practitioners face the same barriers (as academics) – lack of time, lack of expertise, lack of incentive to engage with impact evidence and recommends to add to the paper courses available to impact practitioners.

 I am fully supportive of the idea that research professionals (broadly defined) need greater recognition both within academic and externally. I support the idea that training and professionalization of research impact professionals will help with the academia-to-practice link. However, I struggle to see that a Masters route is the solution to this and can’t see such an offer being appealing to people that I work with or practitioners that we engage with. I think if you are to make a case for a new Masters path there needs to be a stronger argument presented to back it up and clarity about who should participate is such a programme. But for me, of far more value, is the short CPD-type of professional training that is available and I am surprised that the paper has not mentioned organisations such as ARMA, UPEN, Praxis Auril and, for instance, the Canadian Association of Research Administrators, as well as international examples such as Society for Research Administrators International (SRAI), International Network of Research Management Societies (INORMS) etc. (I am sure there are many more international examples). These organisations do offer networking, conferences, briefings and training. No doubt there are gaps and more could be done that brings academics, impact professionals and practitioners together but I don’t really see that gap being filled by a fairly narrowly defined Masters programme.  

 With regard to evaluation techniques and developing impact professional skills in this area, it is worth mentioning that for the most part, when we are looking for evidence of impact, that evaluation needs to be independent and undertaken by an external body, i.e. you can’t be the evaluator of your own impact. 

**Minor corrections required:**

- The affiliations are slightly wrong, I think Mark Reed should be subscript number 1.
- There is a typo in the Plain Language Summary: ‘2inimizin’.
- There is a typo on page 4 ‘4inimizies-competing’.

Is the argument persuasive and supported by appropriate evidence?

Partly

Is the work clearly and cogently presented?

Yes

Is the topic of the essay discussed accurately in the context of the current literature?

Partly

Does the essay contribute to the cultural, historical, social understanding of the field?

Yes

Reviewer Expertise:

I am a specialist in research impact and engagement. My academic background is within the business and management discipline and my research focuses primarily on equality, diversity and inclusion. My research has covered retailing, police and construction sectors. I have also undertaken research on international franchising in retailing.

I confirm that I have read this submission and believe that I have an appropriate level of expertise to confirm that it is of an acceptable scientific standard, however I have significant reservations, as outlined above.

# Reviewer report: Reviewer response for version 2

**DOI:** 10.21956/openreseurope.16766.r37075

**Published:** 2024-02-22

**Authors:** Pedro Gallo

**Affiliations:** 1 Department of Sociology, University of Barcelona, Barcelona, Spain

1. Preliminary comment: It is not an easy task to successfully bring together research impact praxis on one specific area/field of knowledge, let alone do the same for a wider range of sciences, which is what the authors are aiming at. My experience lies only on health and health care impact, and it is from this experience and knowledge that I might have something to contribute with. In this respect, it is worth referring to a compendium by Deeming *et al.* (2017 ^1^) and their analysis of 25 research impact assessment frameworks in health and health care that might be useful to the authors as a reference.

 2. Both the title and the contents of the authors’ work allude to the value of co-creation (between academics and stakeholders/practitioners) in generating research impact. I fully support the idea. In this respect there are at least four pieces of research the authors may want to include/review in their paper:

- Halvorsrud *et al.* (2021 ^2^) systematic review on the effectiveness of the co-creation of research, exploring which are the optimal mechanisms for successful co-creation, which outcomes are of relevance and how power is shared and knowledge generated.
- van Dijk-de Vries *et al.* (2020 ^3^) work on how a co-creative research approach could foster impact by increasing the researchers' understanding of the value of co-creation and the successful engagement of stakeholders in the research project.
- Stier and Smit (2021 ^4^) paper on how co-creation could increase the uptake of scientific knowledge. Some 11 conclusions are drawn including the allocation of adequate resources, a common agenda, reconciling differences in several domains, addressing questions of impact, validation and valorisation from the outset, and so on.
- Greenhalgh *et al.* (2016[ref-5) work enumerating successful principles for co-creation to generate impact.

3. In a number of places in their paper the authors refer to training/capacitation of academics as a successful strategy to enhance the impact of research, to that I agree. I do, however, support this should be part of the culture of the organisations these academics work for, not just training programs, but as part of the set of values, mission and vision (culture) of research organizations to be successful.

 4. The authors mentioned on many occasions the importance of stakeholders. I believe their mapping, identification and involvement in the early phases of research is crucial to fostering impact thereafter. One of the first phases in this process would be the identification of the research needs such academics are willing to respond to. This is a major issue to maximise impact: addressing a real need with stakeholders’ involvement in an early stage.

 5. The points mentioned above indicate that *impact* needs to be addressed *from the design* of the research that is carried out. The authors and reviewers have reflected this idea in the paper, but I believe it is worth stressing that point: impact from design.

 6. There seems to be much to be done on non-academic indicators of impact, that is, on which ways can we appraise impact. Further, it is surely not enough to agree on a given set of indicators but on how impact is really achieved. In this sense, there are many published practices on how qualitative Impact Narratives are being used to capture and enrich that, identifying barriers and facilitators, and connecting individual research cases to context variables. There is very little on this in the authors’ paper, and much less on the difficulty of tracing impact, that is on how to lawfully attribute any plausible impact (on the social, economic, environmental or health domains) to the research carried out.

 7. In their section on “Evidence-based pathways toward research impact” I would include:

- There is a growing need to incorporate research impact as part of the editorial mandates, maybe by means of new formats, methods or editorial priorities.
- There is a need for Communication/Reporting of results to include relevant groups, for example, the gender perspective, minority groups.
- There are no references to barriers and facilitators to the use of the knowledge produced by research. Overcoming such barriers or building on such facilitators are two main strategies to foster the impact of research. Yet these could be very context-specific (previous knowledge, skills, social or professional roles, values and beliefs…), and relate to individual, organisational or systemic levels.

8. In their section on “Key messages”, I find a major point is missing: Creation/stimulus of places for academics and practitioners to interact, work together, and define agendas and facilitators for research impact. Some academics refer to that as push-pull spaces/arenas.

 In brief, I favour the indexing of this paper with some revisions. To my understanding the authors may want to include some additional published work in their paper, and reflect on other relevant points such as impact from design, impact narratives, barriers and facilitators, and academic & stakeholders’’ push/pull spaces.

Is the argument persuasive and supported by appropriate evidence?

Partly

Is the work clearly and cogently presented?

Yes

Is the topic of the essay discussed accurately in the context of the current literature?

Partly

Does the essay contribute to the cultural, historical, social understanding of the field?

Yes

Reviewer Expertise:

Sociology of health, health policy, research impact assessment

I confirm that I have read this submission and believe that I have an appropriate level of expertise to confirm that it is of an acceptable scientific standard, however I have significant reservations, as outlined above.

## References

[1] : Measuring research impact in Australia's medical research institutes: a scoping literature review of the objectives for and an assessment of the capabilities of research impact assessment frameworks. *Health Res Policy Syst* .2017;15(1) : 10.1186/s12961-017-0180-1 22 10.1186/s12961-017-0180-1 28327199 PMC5361798

[2] : Identifying evidence of effectiveness in the co-creation of research: a systematic review and meta-analysis of the international healthcare literature. *J Public Health (Oxf)* .2021;43(1) : 10.1093/pubmed/fdz126 197-208 10.1093/pubmed/fdz126 31608396 PMC8042368

[3] : How to support a co-creative research approach in order to foster impact. The development of a Co-creation Impact Compass for healthcare researchers. *PLoS One* .2020;15(10) : 10.1371/journal.pone.0240543 e0240543 10.1371/journal.pone.0240543 33045033 PMC7549764

[4] : Co-creation as an innovative setting to improve the uptake of scientific knowledge: overcoming obstacles, understanding considerations and applying enablers to improve scientific impact in society. *J Innov Entrep* .2021;10(1) : 10.1186/s13731-021-00176-2 35 10.1186/s13731-021-00176-2 34603927 PMC8475873

[5] : Achieving Research Impact Through Co-creation in Community-Based Health Services: Literature Review and Case Study. *Milbank Q* .2016;94(2) : 10.1111/1468-0009.12197 392-429 10.1111/1468-0009.12197 27265562 PMC4911728

# Reviewer report: Reviewer response for version 2

**DOI:** 10.21956/openreseurope.16766.r34252

**Published:** 2023-08-17

**Authors:** David Phipps

**Affiliations:** 1 Division of Vice-President Research & Innovation, York University, Toronto, Canada

I have no further comments on version 2.

Is the argument persuasive and supported by appropriate evidence?

Yes

Is the work clearly and cogently presented?

Yes

Is the topic of the essay discussed accurately in the context of the current literature?

Partly

Does the essay contribute to the cultural, historical, social understanding of the field?

Yes

Reviewer Expertise:

research impact, knowledge mobilization, institutional research strategy

I confirm that I have read this submission and believe that I have an appropriate level of expertise to confirm that it is of an acceptable scientific standard.

# Reviewer report: Reviewer response for version 1

**DOI:** 10.21956/openreseurope.15318.r28168

**Published:** 2022-03-07

**Authors:** Elyse Aurbach

**Affiliations:** 1 Molecular and Behavioral Neuroscience Institute, University of Michigan, Ann Arbor, MI, USA

The authors have developed a cogent commentary arguing in favor of developing further the field and professionalization of “research impacts.” They note a number of important influences in intersecting systems that connect with research impacts, including funding sources/funders, the relationship with other fields (e.g., science communication), open data trends, etc. They also appropriately note a number of important challenges involved in supporting the evolution of research impacts as a field and describe how these challenges impact the capacity for research impacts efforts to evolve. 

 While I’m wholeheartedly in favor of many of the interventions that the authors propose, I believe that this article would be strengthened by both contextualizing research impacts in a wider view and in diving more deeply into several areas - some ideas outlined below.

 The article would benefit from acknowledging the challenge of *language* around research impacts work, which has myriad other terms (e.g., public impact research, public engagement, public scholarship, engagement scholarship, etc.) and adjacent or intersecting fields (e.g., science communication, public policy, etc.) associated with it. Broadly, one challenge that both scholars and practitioners in this space face is grappling with this challenge in describing the similarities and differences across different forms of work, which inhibits the capacity for like-minded professionals to be able to overcome their respective silos. A critical step that would enable the system to change in the way the authors endorse is to grapple with and address these language barriers. One way of addressing this in the article would be to pull in some of the other germane literatures (e.g., around the role of boundary spanners in higher education, public engagement & public scholarship, and institutional change efforts, etc.) and/or to highlight spaces in which research impacts work are already deeply institutionalized (e.g., the Cooperative Extension system in the United States).

 The authors also state that “scholarship in this domain needs to be more relevant to practice” - while I fully agree with this sentiment, I disagree with the premise that the primary area of scholarship that they propose connecting with practice (i.e., evaluation) will alone be sufficient to create these links. The commentary would be strengthened by acknowledging some of the other, adjacent challenges (and their associated literatures) with connecting research with practice *alongside* evaluation, including: 

1. That there are real, important differences across disciplines with what ‘scholarly rigor’ entails and how this relates to the types of impact goals and efforts most commonly associated with work in those disciplines (e.g., Doberneck & Schweitzer, 2017; Fecher & Hebing, 2021). This challenge is exacerbated when different types of scholarship are valued differently, both within and across fields, which can lead to experiences of epistemic exclusion which differentially impact scholars with marginalized and underrepresented social identities (e.g., Settles *et al*., 2020).
2. Like any groups with differing expertise, researchers and practitioners in research impacts work can - and frequently do - have divergent perspectives on what questions and efforts are valuable to pursue to create a solid evidence basis. As the authors acknowledge, work that will meaningfully move the needle on research-practice linkages will necessarily require that practitioners be involved in setting research agendas, and researchers to be more intimately involved with translating their work to practice. However, funding and other supports for these collaborations are frequently lacking - better training and/or professionalization efforts to support research impacts won’t address the types of resourcing gaps that keep these kinds of partnerships supported on a case-by-case basis.
3. Different forms of expertise are needed at different stages in the research and translation process, and in general, the funding and reward systems in place around research preference discovery and/or novelty over synthesis and translation. The authors might benefit from clarifying *where in the system* they believe that the training and professionalization efforts they propose are likely to have the greatest impact - Is it by creating more research faculty with translation skills? In departments and university units expanding investment in professors of “practice” and other titles related to translation/impact? In deepening a commitment to professional staff to work in partnership with faculty? 

As the authors note, the relationship between systems of higher education and funding are critical levers as related to research impacts. The commentary would benefit from greater clarity about proposed mechanisms to evolve the funding ecosystem, especially in the context of limitations in existing funding mechanisms and allowable expenditures to support engagement and research impacts work (e.g., Can research funds be applied to paying for the effort of professional support staff? Better support the development & scaling of participatory processes?), weighed against real limits in revenue sources & priority in resource allocation.

Is the argument persuasive and supported by appropriate evidence?

Partly

Is the work clearly and cogently presented?

Yes

Is the topic of the essay discussed accurately in the context of the current literature?

Partly

Does the essay contribute to the cultural, historical, social understanding of the field?

Partly

Reviewer Expertise:

public engagement, research impacts scholarship, capacity-building & training

I confirm that I have read this submission and believe that I have an appropriate level of expertise to confirm that it is of an acceptable scientific standard, however I have significant reservations, as outlined above.

## References

[1] : Disciplinary Variations in Publicly Engaged Scholarship: An Analysis Using the Biglan Classification of Academic Disciplines. *Journal of Higher Education Outreach and Engagement* .2017;21(1) :78-103 Reference source

[2] : How do researchers approach societal impact?. *PLoS One* .2021;16(7) : 10.1371/journal.pone.0254006 e0254006 10.1371/journal.pone.0254006 34242271 PMC8270479

[3] : Understanding psychology's resistance to intersectionality theory using a framework of epistemic exclusion and invisibility. *Journal of Social Issues* .2020;76(4) : 10.1111/josi.12403 796-813 10.1111/josi.12403

# Reviewer report: Reviewer response for version 1

**DOI:** 10.21956/openreseurope.15318.r28049

**Published:** 2021-12-10

**Authors:** David Phipps

**Affiliations:** 1 Division of Vice-President Research & Innovation, York University, Toronto, Canada

This paper is a commentary, one designed to stimulate thinking and debate. It achieves this purpose by recommending that "training for research impact work needs to forge strong links between research impact scholarship and practice". This is a sensible recommendation and one that won't find any dispute from me as I am often advocating for exactly that. As with everything the devil is in the details, and I offer thoughts on some of those devilish details below.

 The commentary lacks real world examples of where this is working well to illustrate the principles presented. For example, there is strong impact research and impact practice in the Michael Smith Foundation for Health Research in British Columbia, Canada. For many years MSFHR was led by Bev Holmes, a renowned scholar of impact. My own collaboration as an impact practitioner in Research Impact Canada/York University with impact scholars in the RIPPLE program (Faculty of Education, Queen’s University) are two Canadian examples. There are certainly more.

 Regarding coherent frameworks. I argue that there will likely never be a coherent framework widely accepted and practiced. In contrast I do think there are principles that will translate across many contexts, so I encourage the authors to think of principles instead of (as well as) frameworks.

 Much impact scholarship comes out of discipline specific research. I encourage the authors to differentiate impact scholarship arising from specific disciplines and how practitioners can access and adapt that scholarship to different contexts. And if an impact practitioner is sitting with an institutional mandate, serving many/all disciplines, then how can s/he learn from homelessness, nursing, agriculture, etc. impact scholarship. That being said impact is emerging as it’s own discipline so a reflection on research impact within a discipline and as its own discipline would help point the way forward.

 But then impact practitioners run into the same barriers as other evidence end users: lack of time, lack of expertise, lack of incentive to engage with the impact evidence. I recommend adding to the last bullet under concrete measures, a nod to courses available to impact practitioners to help overcome these barriers. There are a number of courses in Canada (ie Mobilize YU from York University as one of a number) and certain there must be more in the UK and elsewhere that could add concrete examples of efforts to address these barriers. Courses will help bring the impact evidence to impact practitioners.

 In the concrete measures bullet on stakeholder involvement, I think there is a missing element of impact practitioners as the stakeholders of impact scholarship. Impact practitioners are the end users of impact scholarship so a reflection on the unique opportunities for impact scholar/practitioner collaborations (see above) would ground this comment more centrally in the rest of the article.

 I will explain my choice of “partly” for: Is the topic of the essay discussed accurately in the context of the current literature?

- The authors cite their own work in 20 of the 48 references. This is a very high self citation rate, in my opinion. I encourage a broader survey of the literature.
- And at the risk of being self serving (and the joy of open review since no one is blinded to the reviewers), Julie Bayley and I did anticipate this in our 2019 paper in Emerald Open Research in the description of an impact practitioner who can be considered advanced in their research impact literacy. We described this as being “critical”, defined as “ *Critically engaged with the evidence, understands there is a body of expertise, knowledge and tools which can underpin practice and is able to (i) synthesize, (ii) critique and (iii) add to/extend it. Likely to be able to comprehend at a strategic and/or systems level*”. An impact practitioner who is critical is already at the nexus of impact scholarship and impact practice. [https://emeraldopenresearch.com/articles/1-14]

Is the argument persuasive and supported by appropriate evidence?

Yes

Is the work clearly and cogently presented?

Yes

Is the topic of the essay discussed accurately in the context of the current literature?

Partly

Does the essay contribute to the cultural, historical, social understanding of the field?

Yes

Reviewer Expertise:

research impact, knowledge mobilization, institutional research strategy

I confirm that I have read this submission and believe that I have an appropriate level of expertise to confirm that it is of an acceptable scientific standard, however I have significant reservations, as outlined above.

## References

[1] : Extending the concept of research impact literacy: levels of literacy, institutional role and ethical considerations. *Emerald Open Research* .2019;1: 10.35241/emeraldopenres.13140.2 10.35241/emeraldopenres.13140.2
